# Developing Reflection and Collaboration in Translational Medicine Toward Patients and Unmet Medical Needs

**DOI:** 10.3389/fmed.2019.00094

**Published:** 2019-05-03

**Authors:** Moira Clay, Linda T. Hiraki, Lovro Lamot, Basma M. Medhat, Salmaan Sana, Anita R. Small

**Affiliations:** ^1^Moira Clay Consulting, University of Western Australia, Perth, WA, Australia; ^2^Division of Rheumatology, Department of Paediatrics, The Hospital for Sick Children, University of Toronto, Toronto, ON, Canada; ^3^Department of Epidemiology, Dalla Lana School of Public Health, University of Toronto, Toronto, ON, Canada; ^4^Department of Paediatrics, Sestre Milosrdnice University Hospital Center, Zagreb, Croatia; ^5^Department of Paediatrics, University of Zagreb School of Medicine, Zagreb, Croatia; ^6^Rheumatology and Rehabilitation Department, Kasr Al Ainy Faculty of Medicine, Cairo University, Cairo, Egypt; ^7^Better Future, Austerlitz, Netherlands; ^8^Small LANGUAGE CONNECTIONS, Toronto, ON, Canada; ^9^Linguistics, University of Toronto Scarborough, Toronto, ON, Canada

**Keywords:** reflection, team, growth, collaboration, co-creation, transformation

## Abstract

This perspective article aims to highlight the importance of values-driven personal reflection and collaboration for effective translational medicine training. We frame the dilemma in translational medicine and provide an approach for solution emphasizing collaboration and co-creation for innovative change in translational medicine. We cite the science in transition literature suggesting why personal reflection and a collaborative approach is important. We identify the problem with publication pressures and the bibliometric mindset. We focus on motivation to seek and find results that really matter for patients and individuals to maintain health in the real world. We review how the international *EUREKA Institute for Translational Medicine* (established in 2007) works with students, to harness their core values and develop personal growth skills to improve their leadership effectiveness, to work toward collaborative gain and potentially more meaningful results for patients and medical needs. We describe how the *EUREKA Institute's* unique setting, curriculum and hidden curriculum aspects effectively train program participants. The article highlights creating an immersive safe space, personal reflection, connection, structured brainstorming, group problem solving, collaboration and co-creation to facilitate innovation in translational medicine. The article relates program features to their theoretical underpinnings such as Theory U, Mediation Theory and Strategic Innovation Theory. The six authors from different global regions, ages, career stages, translational medicine contexts and years of attendance at the EUREKA Institute provide their reflections on training impact. Lessons learned and recommendations for research and application are discussed.

## Introduction—Dilemma

Translational medicine focuses on the continuum from scientific discovery to its clinical application (1). An increasing number of articles published in prestigious journals address controversies concerning the “crisis in science” and its impact on society, as well as difficulties researchers must overcome in their everyday practice (2–5). It has been suggested that a majority of identified problems, like reproducibility and waste, may originate with publication pressures placed on researchers to assure funding for their endeavors and their career advancement. This tension compels them to forget about real world needs, and to commit themselves to “fashionable science” in order to secure smooth publication. Many scientists in the biomedical field tend to direct their research toward the elucidation of disease mechanism and discovery of new treatment options. While cure is the ultimate goal for every patient, there are also many other needs associated with improving quality of life, that are seldom addressed among researchers of different domains and which someone not spending time with patients cannot fully understand. The dilemma is the widening gap between scientists and patients, as well as between research discoveries and their clinical application. Groups of esteemed and accomplished scientists have therefore created specific guidelines to establish a new scientific system which will have higher impact on health related issues that really matter to distinct communities (6–8). This could mean new promising treatments and/or better quality of life. In order to achieve this, it is important for scientists to strengthen their connections with those whom they plan to serve, such as patient organizations, and to seek out new and non-obvious collaborations among different professionals. It is also essential to change the way research impact and researchers are being measured in order to allocate limited research resources, maximize research benefit and minimize research waste (9–13). Finally, in parallel with large scale changes, the most important initiative and inevitable step toward shedding the narrow bibliographic mindset is personal introspection of every person regarding their role in this theater that we call science. In this article, we describe the process of one translational medicine educational program model. Complementing the recent mixed methods Weggemans et al. study (1), participant reflections in this perspectives article answer, “How has this program had an influence on you and how have you translated that to your work/ personal life?” We will present critical lessons which we have learned from this program model, that provide a means for addressing this crisis in science, including the importance of self-reflection, self-awareness and collaboration to break through siloed thinking and the need for effective engagement across disciplines and between professionals and patients.

## Program Model

The *Eureka Institute for Translational Medicine* certificate program was established in 2007 to educate and build an international interdisciplinary translational medicine community prepared to spearhead application of discoveries that truly improve human health. Funded by university partners, medical journals, and research institutes internationally, it provides an intensive 1-week experience with both content-driven formal curriculum and informal hidden curriculum for practitioners of translational medicine internationally. Researchers such as Hafler et al have identified the hidden curriculum as informal arenas of influence such as unplanned social activities as well as “organizational culture and place that are more invisible and ethereal in their presence and impact” (14). Participants include practicing clinicians, researchers, clinical researchers, basic scientists and industry professionals. *Eureka* partner institutions have a search and selection process focused on selecting faculty members who are investigators and who would benefit from the course and likely to engage in follow up Eureka hub activities locally. Most trainees who attend the course are from the partners; a small number of trainees are selected by the *Eureka* board via an open competition.

Eureka goals are to harness student core values and personal strengths, to work toward collaborative gain and meaningful results for patients and medical needs. Weggemans et al demonstrate that over half of institute alumni report its influence on their commitment to translational medicine. They also report Eureka's influence on how they conduct translational medicine, their engagement with stakeholders and a broader international network that provides a base of support for their translational work (1). “Why translational medicine fails -and what to do about it,” an intensive 1-week course of the UMC Utrecht, Netherlands University Summer school established in 2016, is also an integral component of the *Eureka Institute for Translational Medicine*, reaching international students with an emerging interest in translational medicine early in their careers. The course recruits Ph.D. and postdoc trainees from around the world. Reflections in this article are a result of the authors' experiences as students and faculty in these two related programs of the Eureka Institute. It describes how the unique features and path shared by both these international translational medicine programs present a model to address the challenges faced by the translational medicine community.

## Program Features—Unique Setting

The setting for the *Eureka Institute Certificate Program* is unique with the intention of providing a nurturing and vibrant learning environment for the intense, content driven curriculum and hidden curriculum. Faculty and students interact in a setting of fresco lined vaulted ceilings in a large session space and break out rooms for small group discussion held in the round. The venue is the Borgia Casa tucked away just off the main piazza in Ortigia, Syracusa, southern port and site of ancient ruins and the abode of Archimedes. Sessions are peppered with luscious meals of pasta, fresh market foods, wine, and short breaks with cappuccino or gelato. In the Utrecht, Netherlands Summer school, students meet in an open glass-surrounded classroom, a country estate with break out groups on its immense grounds, or in the modern rooms of a nutrition research science park with beer and bites and dinner along the canal.

The social environment reinforces shared experience, values and beliefs of the formal and informal hidden curriculum. Each physical setting is slightly out of the ordinary creating a unique immersive safe space for personal reflection, connection, structured brainstorming, “out-of-the-box” thinking, group problem solving, collaboration, and co-creation to push the envelope and to facilitate innovation in translational medicine. What is essential to these physical contexts, is creating a positive safe space and protected (uninterrupted) time.

## Program Features—Professional Leadership Curriculum

In parallel with the science content-driven program is the professional leadership curriculum component that moves from personal growth to connection and collaboration establishing an atmosphere of trust. This is an essential first step (15) toward creating collaborations not previously achieved (i.e., *Eureka Institute* certificate program or summer school students with each other, with faculty, patient physician teams, clinical research teams, lab teams, or industry clinical research teams), potentially breaking silos in the competitive translational medicine context.

## Program Features—Instructions and Outcomes

The program does not provide students with specific instructions on how to achieve its goals. Instead, it gives them a unique experience which enables them to identify and understand a problem, think about possible solutions and most importantly, engage in joint activities with other students and faculty. Although outcomes of this approach are difficult to measure, they allow each participant to make the most of their experience gained. Finally, this helps to create a global network providing a context from which real, meaningful, and much-needed changes could arise.

What follows are reflections of the professional leadership curriculum incorporating personal growth and collaboration and its subsequent influence as described by faculty and students of these programs.

## Faculty Reflection, Netherlands—Personal Growth Toward Innovation

Having once upon a time studied medicine and later pursued a career working on change within healthcare, I have wondered and asked myself what role I can play in bringing research to practice? Invited in 2017 to become a part of the faculty of the *Eureka Institute Certificate Program*, I was tasked to find a way for people to connect more with each other, develop their leadership skills and give tools to effect change in their particular setting.

It starts with asking some fundamental questions of ourselves (16): “Who am I?”, “Why do I do the work I do?”, “What does translational medicine mean to me?”, “What gives me both energy and frustrates me about the current landscape of translational medicine?”, and “What do I want to change or have an influence on when it comes to Translational medicine.” As we explore these core questions, there is a model that we utilize to understand the process that “Eurekans” go through during their immersive 1-week study experience. Theory U is a change management process that very naturally and organically can depict how we as individuals and groups go through a process of letting go of our old ways of thinking and opening us up to new perceptions (17). This process starts with learning the art of active listening through facilitated deep conversations, after which the participants are prompted to reflect on both what they said and heard, to understand and empathize with each other and to become aware of challenges that each person faces with respect to Translational Medicine. After this phase of ‘co-creation,” each participant will work on constructive solutions to create a positive change within their own working environment. As a result, Theory U helps in visualizing how we learn to let go of previous preconceptions and patterns, develop a manner of seeing and sensing with a fresh perspective, co-creating with the people we are teaming up with, and allowing a new way of thinking and working to emerge.

## Faculty Reflection, Canada—Collaborative Interaction Toward Innovation

I am an educator and sociolinguist whose work is to connect members of different cultural groups so they are authentically represented in educational and arts institutions (theater companies, museums, TV and broadcast companies) and one of the few non-medical faculty members in the *Eureka Institute Certificate Program* (since 2014) and in the *Eureka Institute Summer School* (since 2016). I have been struck by the similarity in issues facing translational medical researchers with professionals in the arts world wishing to create more effective institutions that not only serve but also grow out of the priorities, interests and values of those whom they serve. Fear and turf protection hamper progress in both the arts and science. Soul searching, building foundations of trust, increased positive relationships, finding shared values, and creative exploration of solutions reliably enhance breakthroughs in problem solving in both the arts and medical science for true institutional transformation.

Theoretical underpinnings of *Eureka Institute* reflection and collaboration include strategic design for innovative change (18) and mediation theory (19). Students create their own personal *Incomplete Manifesto* based on reflections of their core values (18). They discover their communication interaction strengths in different situations based on communication styles questionnaires used by mediators, similar to the *Myers Briggs Type Indicator* (20). Students gain insights on how they approach conflict from the *Thomas Kilmann Conflict Mode Instrument*, strategies for dealing with cross-cultural interactions, conflict, themes that emerge in translational medicine and how to effectively problem solve using constructive negotiation in the arena of shared interests. This allows for breaking through pre-determined positions to generate new shared solutions and to co-create cultural shift for transformations both personally and institutionally.

## Student Reflection, Croatia—Focus on Well-Being

I went to Eureka to better understand and respond to growing skepticism about science in my surroundings, but also in me. During and after my medical education I realized that science, an elaborate system crafted for providing answers which could make a difference, became an aim in itself. Amid these contemporary circumstances, many use scientific research just for their advantage, neglecting its primordial purpose—to enhance well-being for all. Scientific endeavors are often determined by personal interest, and not based on the potential applicability of the results. However, Eureka offered some solutions for these seemingly insurmountable predicaments that are challenging scientists across the globe. This intensive 1-week course has illuminated the wrongdoings that have driven science to this point, and more importantly, has instructed the participants on how to leverage the lost order in which science can make our lives better. Hearing the anecdotes of patients and their families have raised the issue that their needs might often be much more elementary than scientists imagine. Throughout the course, the importance of maintaining a healthy life and work balance was emphasized as one of the most prominent difficulties scientists face on a personal level. Introspective exercises done throughout the course have highlighted the practical value of reconciling inner harmony with everyday strivings. Finally, the time spent together during the formal program of the course, as well as throughout informal gatherings, created strong bonds among participants which are nourished long after the course has ended. After finishing the program, I feel more competent to exploit my merits as a physician-scientist, to commence a meaningful transformation in my vicinity and to contribute to the efforts of a thriving community of responsible researchers that are part of the Eureka Institute global network.

## Student Reflection, Australia—A Journey Beyond Scientific Content

I went to Eureka, as a research strategy expert, curious to understand more about translational medicine and how I could support my organization and its researchers in this vital endeavor.

There was a diverse group of students from all over the world from different disciplines and career stages. We all shared a hunger for driving discoveries into new medicines or diagnostics. There was a similar number of Faculty—all leaders in their field from some of the top institutions involved in translational medicine world-wide. *Eureka* was a “microcosm” of the highly diverse network required to drive translational medicine.

*Eureka* is about the translational medicine journey. The Greek mythology tale of Sisyphus, condemned to an eternity of rolling a boulder uphill then watching it roll back down again, was unfolded during the week to highlight how translational medicine can be an uphill battle. Successfully translating a research finding into a diagnostic or therapeutic tool is getting the right help at the right time from the right people who share your goal of “getting the boulder to the top of the hill.”

*Eureka* taught me that translational medicine is not just about scientific content. It is also about character, connections and self-awareness. Many of the elements of the Eureka continue to influence me, 8 years after my participation. Team building a tent blind-folded under the direction of one team member without a blind-fold showed how a diverse group of people can come together to problem solve and achieve a goal in challenging circumstances. The group exercises, working with peers and two members of Faculty, to develop various elements of the translational medicine process such as building a narrative for funding, were extremely valuable. The journaling aspect of the program was powerful and a practice I continue to this day, as was brainstorming with peers and connecting with an ecology of translational medicine students and Faculty (of diverse backgrounds and disciplines).

## Student Reflection, Canada—New Approaches

*Eureka* has both an explicit curriculum focused on bridging basic science discovery with improved public health, and a hidden curriculum. After my week at *Eureka*, I found myself returning to those informal lessons from faculty and fellow participants, on the link between self-awareness and research success. In our mentoring small groups, we were encouraged to share our personal challenges in establishing our research programs. These were provocative sessions that pushed each of us to reflect and reveal our vulnerabilities, which created opportunities for solutions. It was clear that those challenges were shared across institutions and continents, as were the guiding principles for success.

As an early career MD, Ph.D., in a large, Canadian tertiary care center, I must balance my roles as researcher, clinician, teacher, wife, mother and daughter. Often compartmentalizing time has the unintended effect of placing my personal life at odds with my professional work. Through the *Eureka* sessions and discussions on the impact of individual experience on leadership effectiveness, the shared principles and strategies for successfully managing personal and professional demands became obvious. Reframing my approach, and emphasizing the whole-self, lead to my most vital link between personal growth and professional leadership effectiveness.

When I returned home, I sought ways to foster the optimistic “*Eurekan* spirit” and began making changes in my lab. I engaged each research team member differently, with greater consideration for their individual attributes and goals, hence finding new working connections. I experimented with new teaching techniques in my university classes, and I worked with *Eureka* alumni at my institution to promote the program to a wider audience of those with the drive to pursue further translational medicine training.

Personally, we are encouraging our children to be productive, contributing members of society. Similarly for myself, I am striving to be an agent of positive change, in fostering a scientific community that values collaboration and connection as a means for united success.

## Student Reflection, Egypt—Focus

Perhaps my encounter with *Eureka* is quite different. I am a rheumatologist at Cairo University with a research career far from the lab. Driven by my deep passion for immunology and its intersection with translational medicine, I attended *Eureka* in 2017. Gladly, my learning expectations were replaced by extraordinary concepts embraced by *Eureka*, such as “structured brainstorming” and “hidden curricula.”

Although many of these concepts struck me as pivotal, they are seldom implemented professionally and personally. This is simply because it's not that easy, never was and never will be. Moreover, absorbing these strategies during the intense and bedazzling 1-week experience is one thing, and incorporating them into one's mindset amidst the chaos of our lives and careers is quite another.

Overcoming this challenge is definitely strenuous and elusive yet is a priority to me. It could be achieved through disseminating and applying these paradigm-shifting concepts to our research, careers, fellows, and lives back home; which again brings me to my peculiarity among other *Eurekan* colleagues.

Egypt, a new member of the *Eureka* network, is a country where research is rapidly evolving; with a tighter clinging to dogmatic molds of academia, such as shooting impact factors and numerous publications. Sisyphean and important as these success definitions and aspirations are, they are not the most important. Hence, my role to convey the ultimate principal nurtured by Eureka, which is to focus on constructive and visionary research serving patients' quality of life.

## Summary

A summary of the key personal and program elements impacting the authors' personal evolution to create change in their respective work environments, translational medicine approach and results, can be seen in the key words below. Key words were based on the process we described in this article, namely the journey of participating in *Eureka Institute* programs. Words selected were agreed by author consensus to be most representative of the authors' collective EUREKA Institute experience. They are organized thematically and chronologically to show the development as the program progresses.

**Dilemma**—Skepticism in Science, Crisis in Science, Challenges, Dogma-Mindset
**Program Setting**—Safe Space and Protected Time
**Journey**—Formal Curriculum and Hidden Curriculum—Personal Growth and Team Growth
**Personal Reflection**—Self-Awareness, Whole-Self, Personal Sharing, Let Go of Old Constructs, Purpose of Science Fundamental, Communication Interaction Styles
**Group Process**—Strategic Design for Innovative Change, Strategy
Structured Brainstorming, Communicating, Connecting
Conflict Management, Cross-Cultural Interaction
Shared Goals, Shared Values, Help
Collaboration, Co-Create
**Outcome**—New Ideas Emerge, New “Ways of Doing” Emerge, Reconnection, Transformation

**Figure 1:**
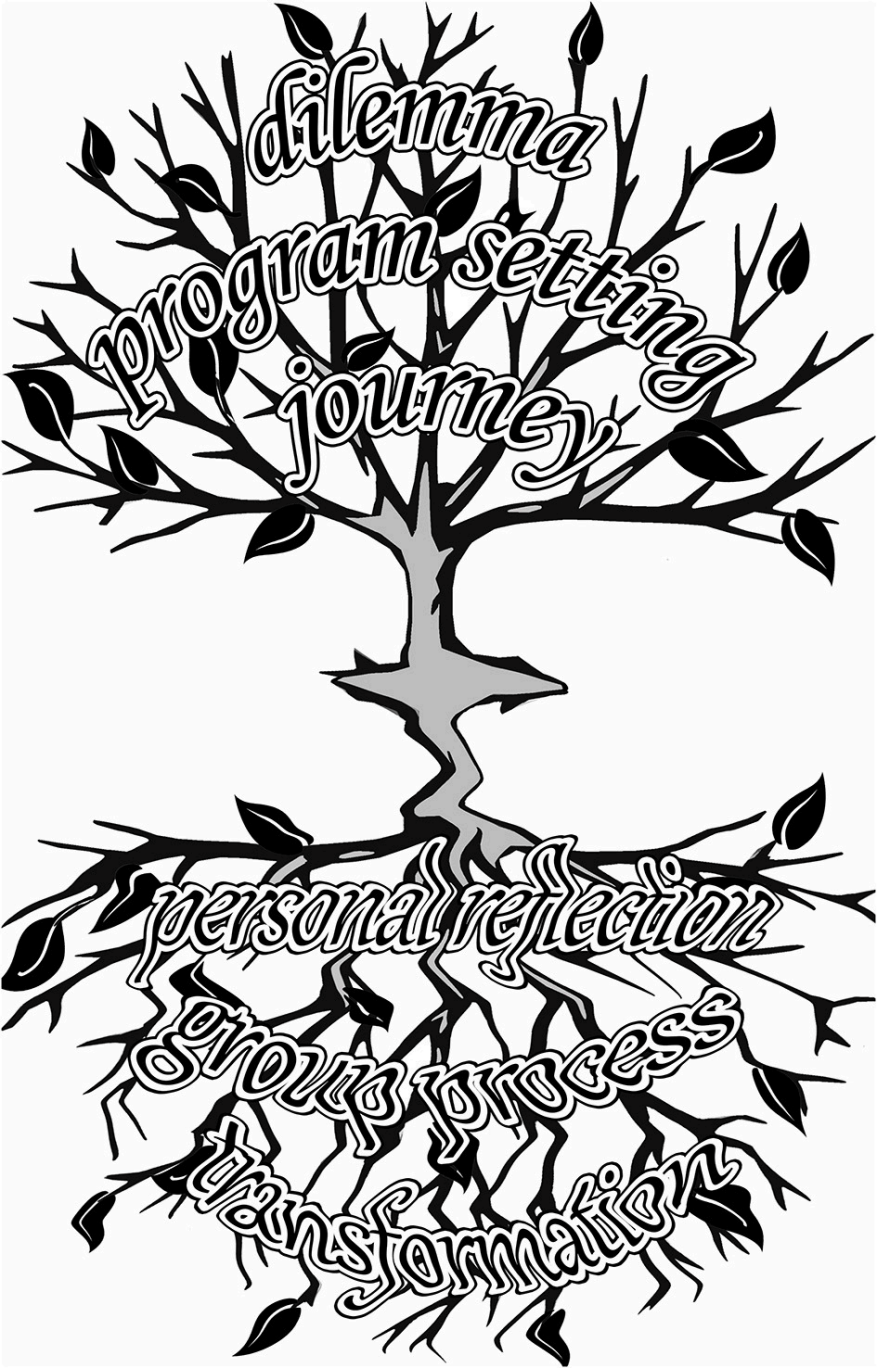
EUREKA Institute - Expansive Experience.

In keeping with *Eureka's* creative approach to problem solving as a way to think outside of the box and expand our perspectives, Figure 1, provides a visual metaphor for personal and team growth rather than a standard logic model of the Eureka Institute Program experience and its impact. The key elements summary is intended to be a descriptive tool while Figure 1 is intended to be inspirational for others wishing to develop similar programs that meet their needs or to attend or create a *Eureka Institute Hub*.

Effective *Eureka* experiences have a synergy between the formal curriculum and the hidden informal curriculum which run in parallel. There is also a synergy between the scientific content components of the Eureka Institute program/s with the personal and team growth approach in the program. The elements integrate like leaves swaying together on branches of a tree. The synergies allow for something expansive to happen leading to a reflection that broadens the picture.

Taken together, the tree and its reflection become a different and more beautiful picture, than that of the tree or its reflection alone.

It's the synergy that makes a learning experience that is both meaningful and impactful on both an individual and collective level. This taken together, creates expansive opportunities for true change in translation medicine, research approaches and outcomes. *Eureka* participants can bring these new reflections and the wholeness of the experience to their lives and work at home.

## Lessons Learned and Recommendations for Future Research and Application

### Lessons Learned

1. The Eureka Institute program creates an environment that demonstrates the importance of self-reflection and collaboration, via exercises anchored in a scientific curriculum.
2. Self-reflection and self-awareness are essential components for effective leadership in translational medicine and research. Knowledge of one's own goals, motivations, biases and limitations, and those of team members and collaborators, positively impacts relationships. This leads to improved communication and productivity.
3. Engaging all stakeholders (i.e., clinicians, researchers, patients, families, industry, and government) at all stages of research, from inception to care delivery, is essential for ensuring the work is relevant and impactful to the emerging medical challenges, that are reflections of patients' needs.

### Recommendations Moving Forward: Call to Action

We recommend identifying and acknowledging your personal motivations for your actions in pursuing translational medicine continually along your journey OR ask yourself, “How can I identify and acknowledge my personal motivations for my actions as I pursue translational medicine?”

Find a context in which you can acknowledge and act upon the personal motivations in yourself as well as in others, in order to strengthen collaborations across disciplines and with patients and connections with the work and process.

Create regular reminders of your core values and discover others' core values to help maintain your priorities, focus and sense of meaningful achievement leading to potentially greater research significance and success in terms of meaningful, impactful health care.

## Author Contributions

AS, SS, LH, MC, LL and BM were all involved in the conception and design of the work and drafted the work or revised it critically for important intellectual content. All authors provided approval for publication of the content and agree to be accountable for all aspects of the work in ensuring that questions related to the accuracy or integrity of any part of the work are appropriately investigated and resolved.

### Conflict of Interest Statement

The authors declare that the research was conducted in the absence of any commercial or financial relationships that could be construed as a potential conflict of interest. The reviewer RGF declared a shared affiliation, though no other collaboration, with several of the authors LH and AS to the handling Editor.
